# Hemispheric dominance in HVC is experience-dependent in juvenile male zebra finches

**DOI:** 10.1038/s41598-024-55987-6

**Published:** 2024-03-09

**Authors:** Sophia Y. Frank, Jesse L. Hunt, Andrea J. Bae, Napim Chirathivat, Sima Lotfi, Sahitya C. Raja, Sharon M. H. Gobes

**Affiliations:** Neuroscience Department, Wellesley College, Wellesley, MA 02481 USA

**Keywords:** Song learning, Lateralization, Zenk, Language learning, Broca’s area, Birdsong, Neuroscience

## Abstract

Juvenile male zebra finches (*Taeniopygia guttata*) must be exposed to an adult tutor during a sensitive period to develop normal adult song. The pre-motor nucleus HVC (acronym used as a proper name), plays a critical role in song learning and production (cf. Broca’s area in humans). In the human brain, left-side hemispheric dominance in some language regions is positively correlated with proficiency in linguistic skills. However, it is unclear whether this pattern depends upon language learning, develops with normal maturation of the brain, or is the result of pre-existing functional asymmetries. In juvenile zebra finches, even though both left and right HVC contribute to song production, baseline molecular activity in HVC is left-dominant. To test if HVC exhibits hemispheric dominance prior to song learning, we raised juvenile males in isolation from adult song and measured neuronal activity in the left and right HVC upon first exposure to an auditory stimulus. Activity in the HVC was measured using the immediate early gene (IEG) *zenk* (acronym for *zif-268*, *egr-1*, *NGFI-a*, and *krox-24*) as a marker for neuronal activity. We found that neuronal activity in the HVC of juvenile male zebra finches is not lateralized when raised in the absence of adult song, while normally-reared juvenile birds are left-dominant. These findings show that there is no pre-existing asymmetry in the HVC prior to song exposure, suggesting that lateralization of the song system depends on learning through early exposure to adult song and subsequent song-imitation practice.

## Introduction

Based on multifarious studies revealing brain asymmetries and lateralization across the animal kingdom^1–4^ and evidence dating back to the Cambrian period^5^, it is believed that lateralization (functional and/or structural) of the nervous system is an evolutionarily old adaptation. The predominant hypotheses explaining why lateralization may be an evolutionary advantage on an individual level (as opposed to population level alignment of biases) are (1) increasing neural capacity and (2) inhibiting incompatible simultaneous responses in both hemispheres^2,4^. Increased neural capacity may be achieved by specializing one side of the brain, which allows for the other side to be utilized for separate additional processes, resulting in parallel processing of information^2^. For example, chicks (*Gallus gallus*) that were simultaneously performing a seed discrimination task and scanning the sky for predators were better at both tasks when they had a lateralized nervous system than when their visual system was non-lateralized due to manipulation of their early visual environment^6,7^. The second hypothesized advantage of lateralization is that hemispheric dominance could prevent simultaneous initiation of incompatible responses to a given stimulus^8,9^. When one hemisphere is dominant over the other (possibly through inhibitory connections with the other side), there is no allowance for incompatible responses initiated simultaneously on both sides of the brain.

Outside of the visual system, examples of hemispheric asymmetry and lateralization of brain function exist in other sensory and motor domains. In humans, part of Wernicke’s area (the planum temporale) is larger in size in the left versus the right hemisphere, and functional left-sided lateralization in Broca’s and Wernicke’s area has been shown to increase as a child develops and learns to speak^10–13^. However, studies in children inherently confound the effects of age and level of experience with the language on development of lateralization. As left-sided hemispheric dominance increases with age, it is correlated with an increased proficiency in linguistics skills^14–16^. It is therefore difficult to determine the extent to which the specific mechanisms of exposure-dependent learning are required for normal patterns of lateralization in the language areas to develop.

**Figure 1:**
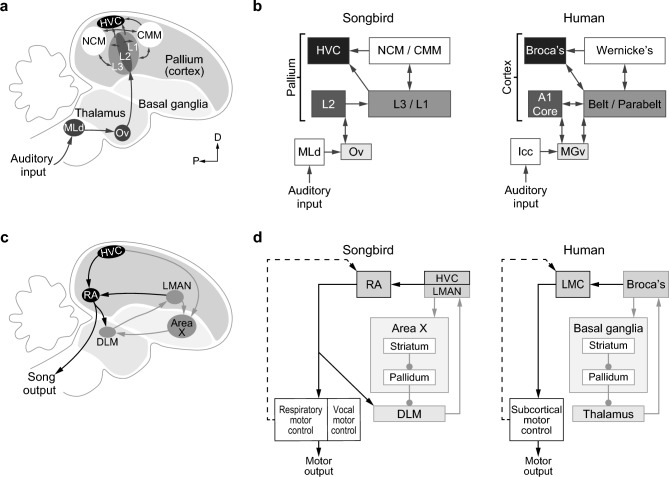
Parallels between human and songbird vocal production and auditory perception pathways. (**a**) Schematic of the vocal perception pathway in songbirds. The primary auditory pathway (dark gray arrows) ascends through a midbrain nucleus (MLd) and a thalamic nucleus (Ov) to the primary auditory region Field L2. L2 relays information to secondary auditory regions L1 and L3, which innervate the NCM and CMM, the higher-order processing regions. These areas provide input to the premotor control center HVC. Modified, with permission, from Chirathivat, et al.^27^. (**b**) Circuit diagrams showing analogous regions in the human and songbird auditory perception pathway. The human auditory pathway also includes a midbrain (Icc) and a thalamic (MGv) nucleus, and the primary auditory cortex (A1) projects to higher-order auditory processing (Wernicke’s) and motor control (Broca’s) areas. Modified, with permission, from Chirathivat, et al.^27^, with additional sources: Bolhuis, et al.^21^, Jarvis^28^. (**c**) Schematic of the vocal production system in songbirds. The primary motor pathway (black arrows) descends from the HVC, to the RA, directly to motor neuron nuclei that control syringeal and respiratory muscles. The anterior forebrain pathway (light gray arrows) is a motor control loop that includes cortical nuclei (HVC and LMAN), basal ganglia (Area X), and a thalamic nucleus (DLM). Modified, with permission, from Brainard and Doupe^29^. (**d**) Circuit diagrams (round arrowheads indicate inhibitory synapses) showing analogous regions in the human and songbird vocal production systems. The LMAN performs an analogous function to Broca’s area in humans, while the RA performs a function similar to the laryngeal motor cortex (LMC). The HVC is functionally analogous to Broca’s area, as it receives auditory input and projects to the primary motor pathway and the anterior forebrain loop^24^. However, comparisons of gene expression profiles and cell-type markers indicate that the HVC may be anatomically homologous to the LMC^28,30–32^. Modified, with permission, from Brainard and Doupe^29^. Abbreviations: *MLd* dorsal part of the lateral mesencephalic nucleus, *Ov* nucleus ovoidalis of the thalamus, *NCM* caudomedial nidopallium, *CMM* caudomedial mesopallium, *Icc* inferior colliculus, *MGv* ventral medial geniculate nucleus, *A1* primary auditory cortex, *DLM* medial part of the dorsolateral anterior thalamus, *RA* robust nucleus of the arcopallium, *LMAN* lateral magnocellular nucleus of the anterior nidopallium, *LMC* laryngeal motor cortex.

Hemispheric dominance of the vocal system is not exclusive to humans^1,8,17–19^. Zebra finches (*Taeniopygia guttata*) show parallels to humans in vocal learning at the behavioral, genetic, and anatomical levels (Fig. 1)^18,20,21^. Zebra finches learn their song in the same way that human children learn to speak, by listening to and imitating adults. For both species, exposure to adults early in development is critical for the emergence of species-specific vocalizations^21–24^. The zebra finch song system also shares anatomical similarities with the human language system, including premotor nuclei, a corticostriatal loop, and a specialized direct connection between the motor cortex and motor neurons of the vocal musculature^25^. The degree of lateralization of neural activity, measured as the expression of an immediate early gene (IEG), in a region analogous to Wernicke’s area in humans, the caudomedial nidopallium (NCM), has been shown to be related to the strength of song learning, just like in humans^26^. Before any exposure to adult song, juvenile zebra finches already show a pre-existing bias to process song with the left hemisphere^27^. This suggests that a small pre-existing bias to process song with the left NCM becomes more pronounced through experience; a process through which hemispheric dominance arises^27^.

We hypothesize that the same experience-dependent lateralization pattern may be present in the HVC (acronym used as a proper name^33^), which is a premotor nucleus in the songbird brain that is part of the primary motor pathway and the anterior forebrain pathway (Fig. 1c)^34–36^. The HVC also receives input from auditory regions (Fig. 1a–b), and thus it is critical in both song learning and song production^37,38^. The connectivity of the HVC, its premotor activity, and its specific role in coordinating timing of song make this region functionally comparable to Broca’s area in the human brain, while recent studies on gene expression profiles and cell lineage have suggested that the HVC may be anatomically more similar to the human laryngeal motor cortex, a primary motor region^28,30,39–41^. Evidence suggests that while the right and left HVC do not differ structurally and are equally active during song production, each side may be responsible for different aspects of song production^40,42^. Interestingly, the HVC exhibits a right lateralized Blood-Oxygen-Level-Dependent signal when birds listen to their own song, while the left HVC is more strongly spontaneously active, measured as increased IEG expression^26,43^.

While we do not know the function of spontaneous molecular neuronal activity in the HVC, we hypothesize that it is related to acquisition either of a ‘behavioral goal memory’, or the motor program for the bird’s own song (BOS) acquired during the sensorimotor learning phase^37,44^. Neuronal, ultrastructural, and behavioral plasticity have been reported after the very first day of experience with song, and the HVC is necessary for acquisition of a behavioral goal memory^37,45–47^. Intracellularly recorded spontaneous (nocturnal) activity is enhanced after a single day of tutoring, and thus molecular neuronal activity is expected to follow a similar pattern, if it is related to the acquisition of a behavioral goal memory^37,45,46^. If that is the case, we would expect to see more molecular neuronal activity right after the first experience with a conspecific song^46^.

To investigate if molecular neuronal activity is triggered by the first exposure to conspecific song suitable as ‘template’ or ‘behavioral goal memory’, and if previous experience with conspecific song (beyond the embryonic and very early post-hatch experience) is necessary for the development of non-singing related lateralization in the HVC, we raised male zebra finches in isolation from adult song. We exposed them to song, rhythmic white noise, or silence, and quantified levels of neuronal activation in the left and right hemisphere to test if molecular neuronal activation is spontaneous or related to song playback.

## Results

**Figure 2:**
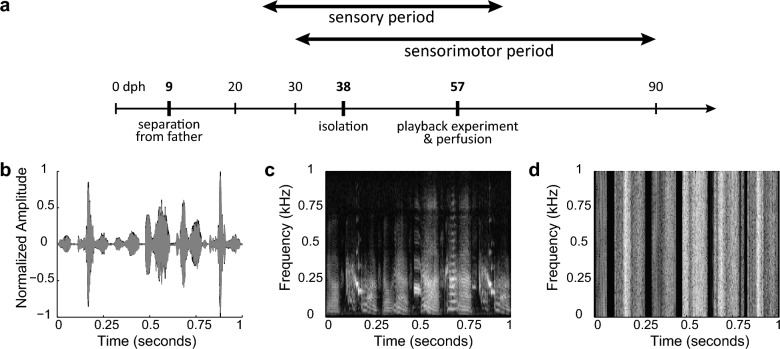
Auditory stimuli for playback experiments. (**a**) Experimental timeline with mean age (dph) at separation from father, isolation, and playback experiment. Playback experiments were performed within the sensitive learning period for normally-reared zebra finches, and consisted of 30 min of playback (silence, white noise, or conspecific song), followed by 30 min of silence before perfusion. (**b**) An overlay of the amplitude plots of the song stimulus (black) and white noise control stimulus (gray), showing matching amplitude and temporal envelope of both stimuli. Spectrograms of the song (**c**) and noise stimulus (**d**). Note the differences in spectro-temporal characteristics.

Juvenile male zebra finches were separated from their fathers at 9 days post-hatching (dph) to isolate them from adult song. Playback experiments were conducted at 57 dph, which is within the sensorimotor learning period in normally-reared zebra finches (Fig. 2a). Juveniles were exposed to one of three auditory stimuli: song of a novel conspecific bird (‘song’), a white noise pattern that was matched to the rhythm and amplitude of conspecific song (‘noise’), or silence (Fig. 2b–d). Birds were then sacrificed and brain tissue was immunohistochemically stained for Zenk (protein product of the immediate early gene *zenk*, an acronym for *zif-268, egr-1, NGFI-a* and *krox-24)*, a marker of neuronal activity. Activity in the HVC was measured as the number of Zenk-immunopositive cells per mm^2^ (density of Zenk+ neurons). HVC lateralization was measured as the difference between left and right hemispheres, divided by the total activation ([L − R]/[L + R]). This ensured that comparisons of lateralization scores were not influenced by differences in absolute activity.

**Figure 3:**
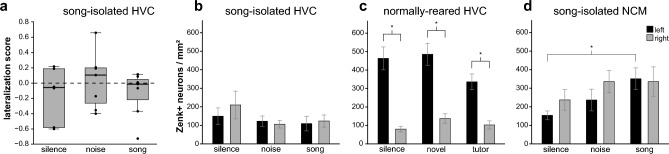
HVC activity is bilateral in song-isolated juvenile male zebra finches. (**a**) Boxplot showing interquartile range (box) and median (black line) of lateralization scores for song-isolated juvenile (57 dph) zebra finches that were exposed to conspecific song (n = 7), rhythmic white noise (n = 7), or silence (n = 6). Lateralization scores were calculated from the number of Zenk-immunopositive neurons per square millimeter in each hemisphere ([L − R]/[L + R]). Lateralization scores were not different from zero for any stimulus group (one-sample t-tests, p > 0.05), indicating that spontaneous HVC activity is bilateral in juveniles raised without an adult song tutor. (**b**,**c**) Neuronal activity (mean number of Zenk-immunopositive neurons per mm^2^) in the left (black) and right (gray) HVC. Error bars represent SEM. Song-isolated juvenile male zebra finches (**b**) show no difference in activity between the left and right HVC (F_(1, 17)_ = 1.228, p = 0.283) regardless of the stimulus presented (F_(2,17) _= 0.758, p = 0.410). For comparison, normally-reared juvenile male (56 dph) zebra finches (**c**) show higher activity in the left HVC than the right HVC (n = 6 silence, n = 7 novel, n = 6 tutor) independent of stimulus exposure (reproduced, with permission, from Moorman et al.^26^). (**d**) Neuronal activity in the left and right NCM. Error bars represent SEM. Song-isolated juvenile male zebra finches show a significant difference in activity between stimuli in the left (F_(2,15)_ = 4.989, p = 0.023; song > silence, p = 0.022), but not in the right NCM. (* indicates p < 0.05).

A repeated measures ANOVA with hemisphere as a within-subjects factor (left, right) and stimulus group as a between-subjects factor found no main effect of stimulus (F_(2, 17)_ = 0.758, p = 0.410) or hemisphere (F_(1, 17)_ = 1.228, p = 0.283) and no significant interaction between hemisphere and stimulus (F_(2, 17)_ = 0.691, p = 0.515; Fig. 3b). Within each stimulus group (song, noise, silence), we found that lateralization scores were not significantly different from zero (song: t_(6)_ =  − 1.182, p = 0.282; noise: t_(6)_ = 0.244, p = 0.815; silence: t_(5)_ = − 1.003, p = 0.362; Fig. 3a), indicating that there was no hemispheric dominance in HVC activity. To test whether the lack of lateralization in these birds is a result of low spontaneous molecular neuronal activity in both hemispheres, or due to failed immunohistochemistry, we analyzed *zenk* expression from the lateral NCM from these same animals, which was previously published as part of a larger study^27^. A one-way ANOVA revealed that *zenk* expression in the left NCM significantly differed among stimulus groups (F_(2,15)_ = 4.989, p = 0.023; Fig. 3d). Bonferroni post-hoc tests demonstrated that *zenk* expression was significantly higher in birds that heard song playback over silence (p = 0.022). Thus, song-isolated juvenile male zebra finches show bilateral activity in the HVC, regardless of auditory exposure, while auditory stimuli did evoke stimulus-driven *zenk* expression in the lateral NCM.

Singing is known to induce activity in the HVC^48^. As several (n = 11) birds across the three groups sang during stimulus exposure, we tested if the amount of singing during playback affected *zenk* expression in the HVC. We found a positive correlation between the cumulative duration of singing (in seconds) and *zenk* expression in the right HVC (r = 0.758, n = 9, p = 0.018), but not in the left HVC, and neither did song duration affect HVC lateralization scores. Overall, *zenk* expression levels did not significantly differ between singing and non-singing birds (t = − 1.585, n = 20, p = 0.144), and the significant correlation in the right HVC seemed to be driven by three animals that produced much more song (> 200 s) than the other six birds (birds that produced > 0 s and < 30 s song: r = 0.06, n = 6, p = 0.907). To ensure that the observed bilateral HVC activity was not a consequence of song production, we removed the birds that sang during stimulus presentation for further analysis. In juvenile males that did not sing during stimulus presentation, there was no effect of stimulus (F_(2, 7)_ = 0.157, n = 10, p = 0.857), or hemisphere (F_(1,7)_ = 0.002, p = 0.969), or interaction between them (F_(2, 7)_ = 0.184, p = 0.836). HVC lateralization scores were also not significantly different from zero (p > 0.05 for all groups).

## Discussion

In order to understand the ontogeny of lateralization of vocal control regions, we examined the effect of isolation from exposure to adult vocalizations on the pattern of lateralization in the HVC of juvenile zebra finches. We found that molecular neuronal activity, measured as the expression of the immediate early gene *zenk*, in the HVC of juvenile male zebra finches is bilateral when raised in the absence of adult song. These results suggest that prior to song learning, spontaneous HVC activity is not lateralized, in contrast to the left-dominant spontaneous activity observed in normally-reared juvenile males (Fig. 3c)^26^. The HVC is a premotor area in which molecular neuronal activity is induced by singing and not by playback of auditory stimuli in both normally-reared juvenile and adult zebra finches^26,44^. Although we hypothesized that the first exposure to adult song could initiate a molecular neuronal response in the HVC, we found no increase in molecular neuronal activity in the HVC of isolation-reared birds that were briefly exposed to a conspecific song stimulus. This indicates that activity is not driven by song playback, consistent with normally-reared juveniles and adults. However, in contrast to normally-reared juveniles, which show heightened spontaneous *zenk* expression in their left HVC, *zenk* expression in the HVC of isolation-reared zebra finches is not lateralized during the period that would normally have been their sensorimotor learning period (45–69 dph).

It is not clear how spontaneous left-dominant molecular neuronal activity in normally-reared birds relates to song learning. In the HVC, spontaneously firing neurons are either local inhibitory interneurons or Area X-projecting neurons, rather than RA-projecting premotor neurons^49^. In the NCM, song-induced *zenk* expression was found mostly in inhibitory neurons^50^. Thus, it is possible that the Zenk-positive cells quantified in the current study are local inhibitory interneurons of the HVC. When listening to tutor song, interneurons in HVC are more active during well-learned syllables, inhibiting premotor activity and minimizing further plasticity^51^. As song learning progresses, the firing patterns of HVC interneurons become more temporally aligned to the tutor song template^51^. The activity of HVC interneurons is therefore closely linked to song learning, and learning-induced changes in firing patterns could result in changes in spontaneous expression of *zenk* or vice versa.

Our data were collected in juveniles, with the majority not singing much during the playback experiment, and thus we cannot draw conclusions about lateralization of the song system during song production. Electrical stimulation of the HVC in singing adult zebra finches suggests that HVC activity during singing is bilateral and highly coordinated across hemispheres^52^. It is possible that the specific neurons targeted by these methods are a different subset of HVC neurons than the spontaneously active neurons we observed. The neurons affected by electrical stimulation may be premotor projection neurons, which sparsely fire during song production and are temporally locked to specific syllables^53^. The (lack of) spontaneous molecular neuronal activity we observed may be related to mechanisms of learning and plasticity, processes which are known to induce IEGs such as *zenk*^54^. Despite spontaneous molecular neuronal activity being lateralized in juveniles that have imitated (part of) their tutor’s song, in singing juvenile zebra finches *zenk* expression is bilateral, indicating that there is no hemispheric dominance during song production^55^.

The absence of lateralization of the HVC in the isolation-reared birds in our study could alternatively be explained by delayed maturation. In isolation-reared birds the sensitive period is extended well into adulthood, and song crystallization does not happen until a much older age^56^. Since we only measured lateralization in the sensorimotor learning period, perhaps a left-lateralized pattern of spontaneous neuronal activity in HVC would have appeared closer to the age in which song isolates experience crystallization of their songs. Although it is unlikely that lateralization would naturally happen at a later age in song-isolates, especially because in normally reared juveniles it is already present at ~ 56 dph, follow-up studies are needed to determine if the HVC can eventually become lateralized even without exposure to an adult tutor. Additionally, isolation-reared birds were deprived of social interaction with an adult male from 9 dph onwards, and spent about 3 weeks in complete social isolation while they were practicing their own ‘isolate’ songs (from ~ 38 to ~ 57 dph). The socially reared birds in Moorman et al.^26,57^ stayed with both parents until 47 dph, after which they were transferred to group housing with their male siblings, and spent two nights and one day in social isolation prior to song playback experiments. While it is likely that the lack of experience with a suitable song model and/or imitation success (resemblance between the song model and the bird’s own song^44^) is driving the differences in HVC lateralization, we cannot exclude that differences in other aspects of social interaction have had an effect as well.

A similar lateralization pattern of neuronal activation in song-isolated versus normally-reared birds has been observed in the auditory processing region NCM. Normally-reared juveniles showed left-dominant activity in the NCM^26^ when re-exposed to the song of their tutor, while song-isolated juveniles did not show any hemispheric dominance when first exposed to song^27^. Thus, like the HVC (this study), experience with a song tutor in the sensory phase of development affects molecular neuronal activity in the left NCM, through which left-hemispheric dominance arises. Unlike in the HVC, activity in the left NCM was modulated by auditory stimuli in both song-isolated and normally-reared juveniles. Perception of conspecific song (first or re-exposure to tutor song) induced more *zenk* expression in the left NCM as compared to silence. Stimulus-modulated activity may be related to auditory memory formation in the NCM^57^, while emerging left-lateralization in HVC, a premotor region, could be related to maturation of the song production pathway and driven by song-imitation practice. Consistent with this idea, *zenk* expression in the HVC of adult males while listening to the bird’s own song is correlated with fidelity of song imitation^44^.

In this study, we sought to understand the primary driving force, either inherent asymmetries or learning-dependent lateralization, behind the development of a lateralized nervous system. In both the NCM and HVC, the molecular neuronal activity seen in normally-reared birds versus song-isolated birds was consistently higher in the left hemisphere than the right hemisphere. This suggests that while left dominance of molecular neuronal activity of these brain regions is not present before experience with adult song, the left HVC and left NCM have an underlying predisposition to be modulated more strongly by experience than regions in the right hemisphere. Our results in zebra finches provide an example in which exposure to adult vocal models during a sensitive period of learning is required for normal lateralization to develop. This supports the hypothesis that lateralization arises as the result of left-biased learning mechanisms, and not by simply utilizing pre-existing asymmetric systems^58^. While some regions in the human brain involved in language become more lateralized during development, future studies could reveal if, like in songbirds, exposure to adult speech early in development is a prerequisite for the emergence of functional lateralization.

## Materials and methods

### Subjects

Twenty one male juvenile zebra finches were reared in the animal facility of Wellesley College (Wellesley, MA) on a 16:8 light:dark cycle. At 9 (± 1.5 SD) days post-hatching (dph), each clutch of juveniles was removed with their mother from the aviary and housed in an acoustically isolated cage to prevent exposure to adult song. At 38 dph (± 4.6 SD), juveniles were transferred into individual acoustically isolated cages. All birds were provided seed, grit, and water ad libitum. Normally-reared birds were raised in breeding cages with their parents and siblings until 47 dph (see Gobes^57^ for details). Experiments were performed between 45 and 69 dph, equivalent to the plastic song stage in normally reared birds Immelmann^59^; Johnson et al.^60^; mean age at the day of experiment was 57 dph (± 7.8 SD). Experimental procedures were in accordance with United States law and approved by the Institutional Animal Care and Use Committee of Wellesley College (IACUC #1106). The study is reported in accordance with ARRIVE guidelines.

### Experimental procedures

One day prior to stimulus exposure, birds were transferred to a sound-proof chamber equipped with a microphone and a speaker for acclimatization; light cycle was maintained. On the day of the experiment, lights were turned on manually for the duration of the stimulus exposure to ensure birds were awake. Stimulus presentation started between 10:00 and 11:00 AM and lasted 30 min. Birds were sacrificed 30 min after the last stimulus presentation. The birds were kept in darkness and silence for the 30 min post-stimulus period to prevent them from vocalizing and thus evoking activity-based molecular neuronal activation. Subjects were exposed to one of three treatments: a recording of the normal song of an adult male zebra finch (Song; n = 8; n = 7 for lateralization data as sections from one right hemisphere were damaged), a rhythmic white noise stimulus (Noise; n = 7) with the same temporal envelope as song and of equal amplitude and duration (Fig. 2b–d), or silence for the control group (Silence; n = 6). Song and Noise groups differed only in that the Song group heard a stimulus that contains species-specific spectrotemporal characteristics. Animals were allocated randomly over the groups, except for birds from the same nest which were non-randomly assigned to different groups. Sample size was determined by the previously published data set^27^, with only males included in the current study as females do not sing. Stimuli were broadcast through a speaker using Windows Media Player to control sound pressure level at 65 dB mean SPL at 30 cm from the speaker. Birds were recorded throughout the experiment to ensure that the birds were awake during stimulus presentation and to monitor vocal activity during exposure. An observer also monitored vocal activity in real-time by following the recording application’s output screen and counting the number of song bouts and calls made by the experimental bird.

### Tissue collection

One hour after stimulus onset, subjects were anesthetized with 0.03 mL Natriumpentobarbital (intramuscular) (Fatal Plus, Vortech Pharmaceuticals, Dearborn, MI) and subsequently perfused with phosphate buffer (PB, pH 7.4) containing 0.2% heparin, followed by fixation with 2% paraformaldehyde and 0.075% gluteraldehyde in PB. Whole brains were dissected out, separated by hemisphere, and post-fixed at 4 °C in 2% paraformadehyde and 0.075% gluteraldehyde in PB for 4 h. Parasaggital sections (50 μm) were made on a vibratome and stored in PB overnight at 4 °C or in cryoprotectant at − 18 °C.

### Immunocytochemistry

Sections were rinsed three times in PB (5 min each rinse) and incubated in H_2_O_2_ (0.03%) for 8 min. Sections were rinsed three times with phosphate buffered saline (PBS) and 0.01% BSA-c (acetylated bovine serum albumin; BSA-c, Aurion, Wageningen, the Netherlands) for 10 min each and incubated with 5% normal goat serum (NGS) in 0.01% BSA-c for 30 min. Afterwards, sections were rinsed three times in PBS and 0.01% BSA-c for 5 min and incubated with primary polyclonal rabbit antiserum (Santa Cruz Biotechnology, Santa Cruz, CA; Cat. No. sc-189, 1:1,000; immunocytochemistry was performed before the antibody was discontinued) raised against the carboxy-terminus of mouse *egr-1* (sequence STGLSDMTATFSPRTIEIC; see Mello and Ribeiro, 1998^61^) and 0.01% BSA-c overnight. Sections were rinsed again in PBS 10 times for 5 min, incubated with biotinylated goat anti-rabbit (IgG, dilution 1:500, Vector Laboratories, Burlingame, CA), for 1 h (room temperature [RT]), and rinsed three times for 15 min in PBS. Afterwards, sections were incubated (RT) with ABC (avidin-biotinylated enzyme complex, Vector Elite Kit, Vector Laboratories) and rinsed in PBS twice for 5 min. Finally, sections were incubated in diaminobenzidine medium with 0.034% H_2_O_2_ for 4 min (RT). The reaction was stopped in distilled water. Sections were then rinsed in PBS, mounted on slides, dehydrated, and embedded in DPX-mountant (RT). Birds of different experimental groups were run in parallel with one another on the same well plate and controls were performed for which the primary or secondary antibodies were omitted. All procedures were performed cold (4 °C) unless otherwise specified.

### Image analysis

Quantification of Zenk-immunopositive cells was performed for HVC on 306 × 562 μm images of three independent sections per hemisphere. Data from NCM were collected from a previously analyzed dataset [Chirathivat et al. 2015]. Digital photographs were taken using a SPOT Insight 2 Mp camera (SPOT Imaging Solutions) and the QCapture 2.9 program (Quantitative Imaging Corporation) on a Nikon Eclipse 50i (Nikon Instruments) with 20 × objective. Image analysis was carried out with a PC-based system equipped with Image J (NIH, Bethesda, MD), and only cells visible along the top surface of each section were counted (excluding the z-axis), to allow for a comparison with the data by Chirathivat et al.^27^ and Moorman et al.^26^. The density (cell counts / area) of three sections per hemisphere per animal were averaged for further statistical analysis. Image analysis was performed ‘blind’ to the experimental history of the subject. No birds were excluded from the dataset; however, some sections got damaged during processing resulting in missing data points.

### Behavioral analysis

The number of calls and song bouts were initially counted by a live observer. Recordings were then analyzed using Praat software^62^ in order to quantify vocal activity, as singing is known to affect *zenk* expression in the HVC^48^. Files containing songs of the experimental bird were identified by visual examination. The number of files containing song bouts were counted, and song duration was measured as the cumulative length of all song bouts. The number of calls made by the experimental bird during the stimulus exposure were manually counted through inspection of the recordings. In case of equipment failure resulting in loss of the recordings, the real-time counts for songs and calls were used (n = 1).

### Statistical analysis

Densities of Zenk-immunopositive cells were natural log-transformed before statistical analysis. A repeated measures ANOVA with within-subjects factor ‘hemisphere’ (left, right) and between-subjects factor ‘stimulus’ (song, noise, silence) was conducted to assess the effects of the auditory stimulus on neuronal activation in the HVC and lateral NCM (data from the same male subjects; previously published in Chirathivat et al.^27^). Bonferroni post-hoc tests were used if needed. Lateralization scores were calculated by dividing the difference in *zenk* expression between the two hemispheres by the total amount of *zenk* expression of the two hemispheres: [L − R]/[L + R]. One-sample t-tests were performed to assess if lateralization scores were different from zero (i.e., activity was bilateral). To examine the effects of vocal activity on *zenk* expression in the HVC, we calculated bivariate correlations between the number of calls, number of song bouts, duration of song, and left HVC *zenk* expression, right HVC *zenk* expression, and lateralization scores. We then conduced an independent samples t-test to determine if there was a difference in *zenk* expression between birds who did and did not sing during playback. Data were analyzed using SPSS 28.0.1.1 (IBM Corporation).

## Data Availability

The datasets generated during the current study are available from the corresponding author on reasonable request.
